# A rare case of self-injection of elemental mercury

**DOI:** 10.1186/s13104-016-1992-8

**Published:** 2016-03-25

**Authors:** Singankutti Mudalige Thanuja Nilushi Priyangika, W. G. S. G. Karunarathna, Isurujith Liyanage, Methsala Gunawardana, Buddini Dissanayake, Sumeda Udumalgala, Chamith Rosa, Thilina Samarasinghe, Pravin Wijesinghe, Aruna Kulatunga

**Affiliations:** National Hospital of Sri Lanka, Colombo, Sri Lanka

**Keywords:** Self-injection, Elemental mercury, Mentally sound, Chelation

## Abstract

**Background:**

Self-injection of elemental mercury is a rare finding especially in healthy people who are mentally sound. Early detection and removal of mercury from the body by chelation and physical removal of a stored injected site is required to prevent long term toxicity.

**Case presentation:**

A 15 year old previously healthy girl presented with an acute febrile illness with a generalized maculopapular skin rash for 3 days with a preceding history of self-injection of mercury to both her forearms. This was an imitating experimental act influenced by a movie and she was mentally sound. Very high whole blood mercury levels, x-rays of the forearms and histology confirmed mercury poisoning.

**Conclusion:**

Self-injection of elemental mercury can also occur in mentally sound people and rapid diagnosis and decontamination is required. This also signifies the importance of imposing limitations for visual media which could misguide minors and lead those to imitate and cause serious self-harm.

## Background

Mercury is the only metal that exists in liquid form at room temperature. It has no essential biological function. It is used in the manufacture of switches, thermometers, sphygmomanometers, extraction of gold and silver and also known to be used in preservatives, pesticides and tooth fillings [1]. Exposure to mercury can cause mercury poisoning which is also known as hydrargyria or mercurialism. Exposure can be inhalation, ingestion or injection. Self-injection of mercury is very rare but is well documented [2]. We report a case of deliberate self-injection of mercury by a 15 year old girl, inspired by a movie who subsequently achieved complete recovery following removal of mercury by chelation and tissue exploration of the injected sites.

## Case presentation

A 15 year old previously healthy girl was brought to our acute medicine unit by the parents with a 3 days history of fever and a generalized maculopapular, non-itchy rash. On further questioning the parents revealed that she had injected 2 ml of mercury by herself to both the antecubital fossae. This was a deliberate action which she had done 1 week prior to the admission and denied any suicidal thoughts. It was an imitating, experimental act influenced by a movie which gave the wrong impression that injection of liquid metals to the bones can convert bone to metal. She did not give similar occurrences in the past and had good school performances and a normal IQ (intelligence quotient). On general examination she was febrile with a generalized maculopapular, non-scaly rash and mild inflammation at injection sites was noted. There was no lymphadenopathy. Rest of the clinical examination was unremarkable.

**Fig. 1:**
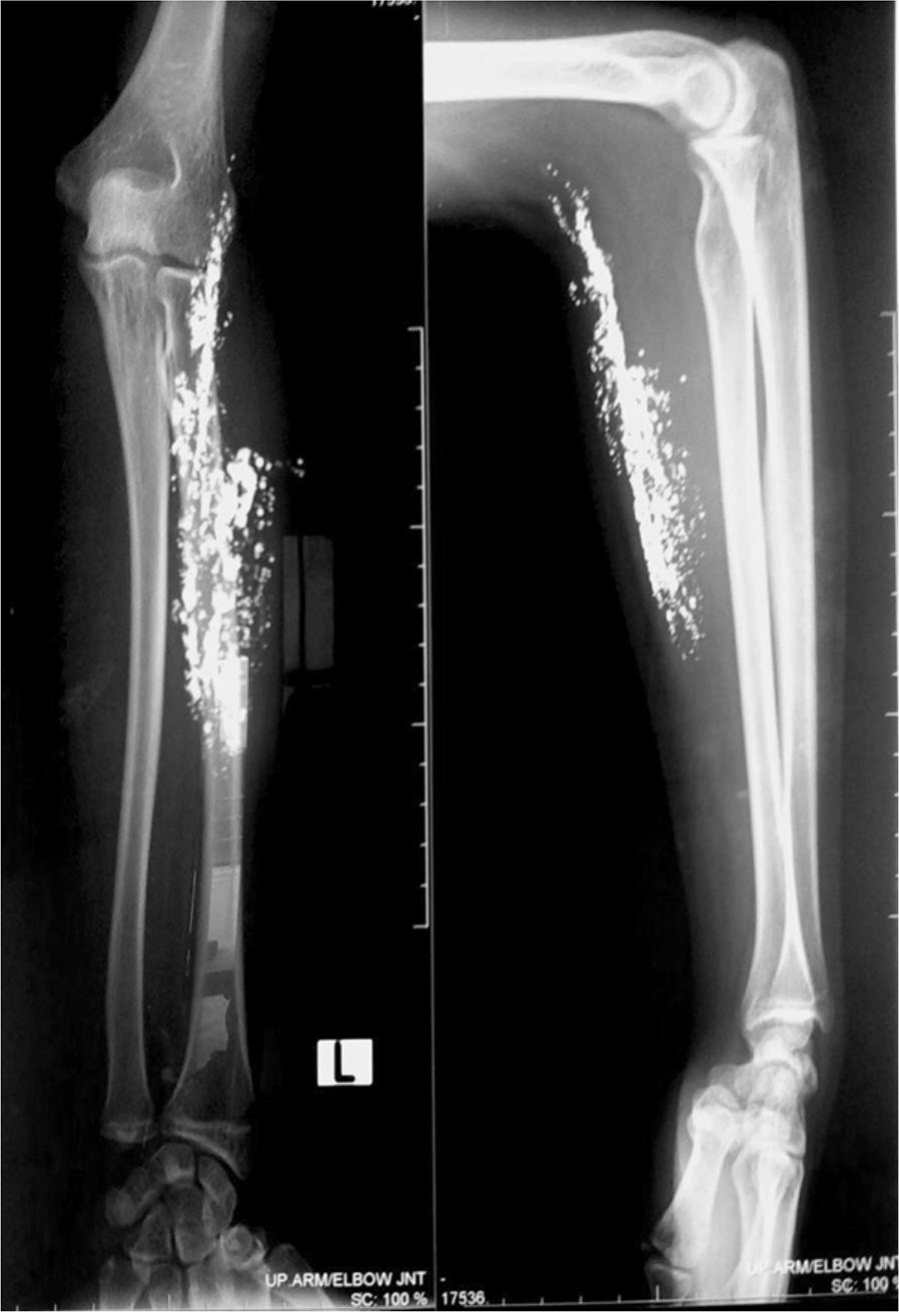
x-rays of hands showing deposition of mercury in subcutaneous and deep tissues

Her x-rays of both forearms revealed subcutaneous depositions which are compatible with mercury (Fig. 1). Whole blood mercury levels was 183.2 μg/L (normal range 0.46–7.5). All other basic investigations were normal.

**Fig. 2:**
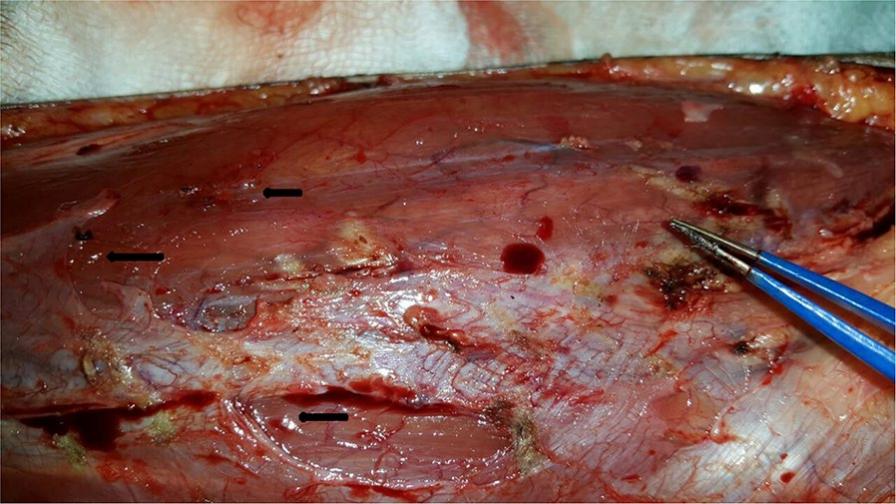
*Arrows* show subcutaneous deposition of mercury

**Fig. 3:**
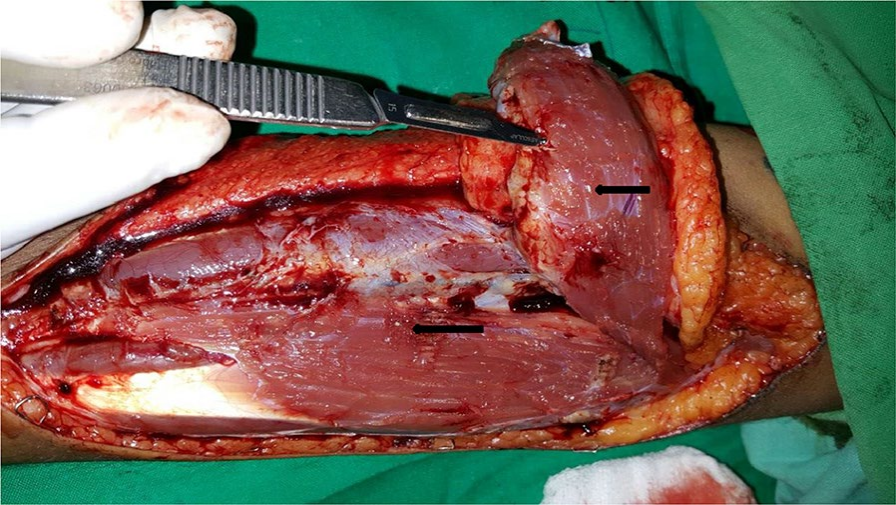
*Arrows* show deposition of mercury inside the muscles

**Fig. 4:**
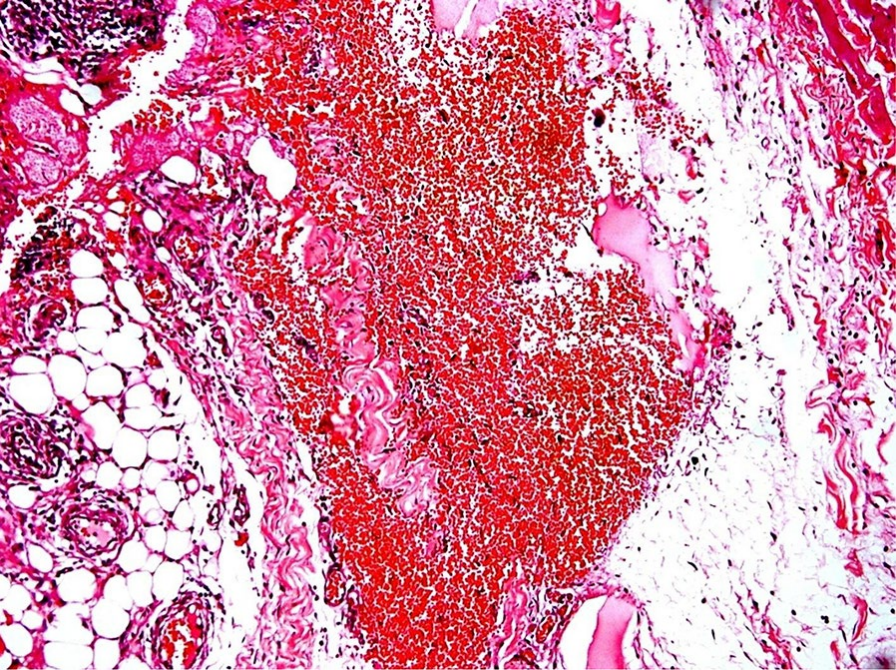
H and E staining of the subcutaneous tissues showing mercury droplets and micro abscess formation

**Fig. 5:**
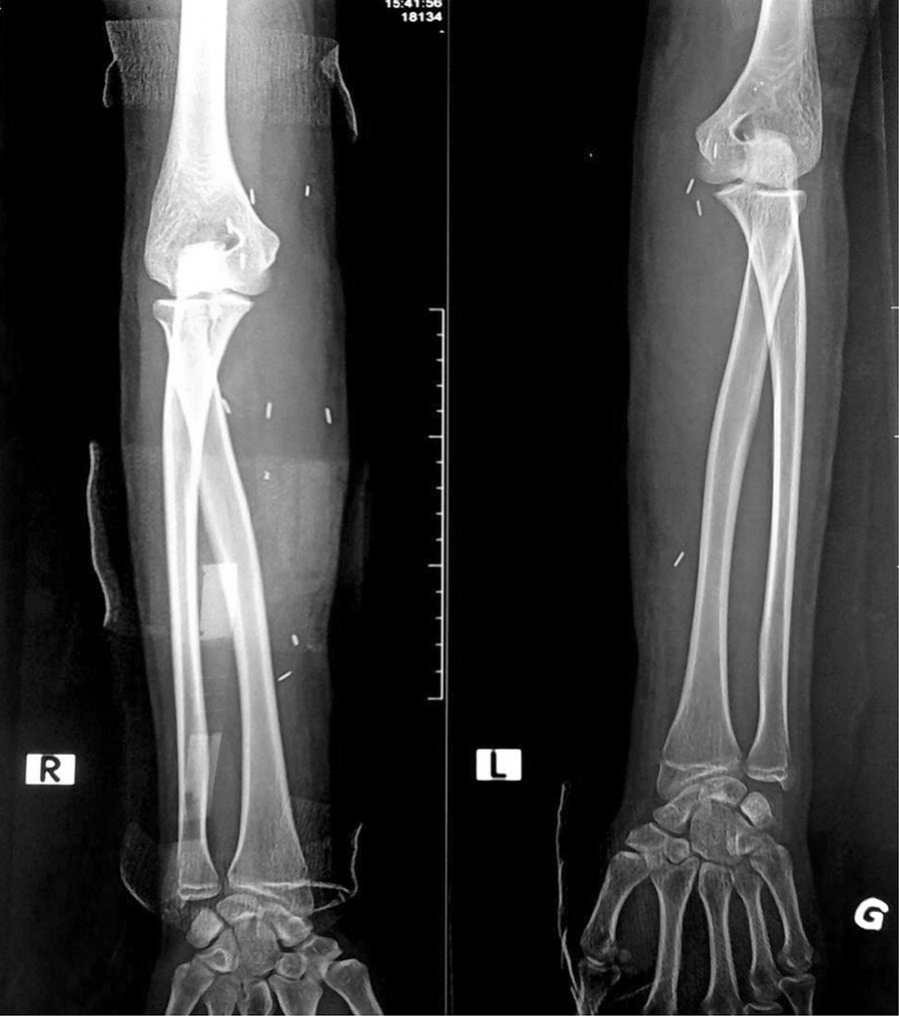
post-surgical x-rays of hands showing full clearance of mercury. (Plaster casts and surgical clips also visible in this x-rays)

She was treated with intramuscular (IM) dimercaprol 3 mg/Kg 4 hourly for 2 days, 6 hourly on 3rd day and then once daily for 3 weeks. At the same time physical removal of mercury was arranged by wide margin excision down to muscle in both her forearms. During surgery depositions of mercury were identified (Figs. 2, 3) and histology (Fig. 4) further confirmed it. She recovered uneventfully and whole blood mercury levels reduced to normal after 4 weeks. Post-surgical x-rays revealed clearance of mercury (Fig. 5). She was revived 2 weekly for 1 month and then monthly for another 6 months. Her whole blood mercury levels were remained normal during this period.

## Discussion

### Mercury exists in three forms

1. Elemental mercury—silvery, shiny volatile liquid which gives off colourless, odourless vapour at room temperature.
2. Inorganic salts—when elemental mercury combines with other elements to form mercury salts.
3. Organic compound—when elemental mercury combines with carbon such as in methylmercury.

In any of these forms, it is poisonous. Exposure can occur in many ways such as inhalation of its vapor, ingestion and intravenous injection [3]. Organic compounds of mercury are more toxic than elemental, or inorganic salts of mercury, and inorganic salts of mercury are more toxic than elemental mercury. Inhalation of elemental mercury vapors are more hazardous than its ingestion or intravenous injection [3]. Organic compounds of mercury especially methyl mercury are concentrated in the food chain (biomagnification). Fish from contaminated water are the main source. The classic example is Minimata disease which occurred in the residents around the Minamata bay in Japan [4]. Mercury was also an essential part of many different medicines in the past such as diuretics, antibacterials and antiseptics. At present mercury is used in tooth fillings, thermometers, sphygmomanometers and in vaccines [5]. In our case the patient’s father who is a science enthusiast, had stored little amount of mercury at home for his experiments. According to the father, one of his friends has given this mercury to him but he denied to reveal further information regarding the source.

Mercury toxicity also occurs in various other ways depending on the form of mercury, the amount of exposure, and the route of entry into the body. It most commonly affects the neurological, gastrointestinal and renal systems. Symptoms of mercury toxicity are manyfold and it can be acute, subacute or chronic. Patients can present with numbness and tingling of peripheries, hearing loss, visual difficulties, gait unsteadiness, tremulousness, desquamating skin rash and emotional and cognitive difficulties [6–8]. Self-subcutaneous injection of elemental mercury is rare and generally does not lead to systemic effects [2]. Abscess formation is the most common local presentation in patients with self-injection of mercury [3]. Our patient presented with a maculopapular skin rash and histology revealed microabscess formation.

The main mechanism of mercury toxicity is due to the irreversible inhibition of selenoenzymes, such as thioredoxin reductase which restores the antioxidant molecules back to their reduced form [9, 10]. As such, the cells which have a high oxygen consumption rate like brain tissues are particularly vulnerable.

Mercury toxicity should be diagnosed quickly and decontamination started by removing clothes, washing skin with soap and water followed by chelation and physical removal as in a case of injection. Chelation of acute mercury poisoning can be done with DMSA (dimercaptosuccinic acid), DMPA (2,3-dimercapto-1-propanisulfonic acid), dimercaprol [British anti-Lewisite (BAL)] or D-penicillamine (DPCN) [11]. DMSA is given orally and it has few side effects and is superior to DMPA, BAL or DPCN [11]. Evan though N-acetyle cysteine (NAC) and glutathione (GSH) were used in the past, evidence suggests that they can be counterproductive [12]. In some studies zinc and selenium have been shown to exert a protective effect most likely by induction of metal binding proteins, such as metallothionein and selenoprotein [12], but some found little evidence for this [13].

The duration of chelation depends on the serum mercury levels, and should be continued till the levels become normal.

Our patient presented with acute mercury toxicity with high serum mercury levels. x-rays of the hands and histology confirmed this. Chelation was started with intramuscular dimercaprol and at the same time surgical exploration and removal of tissues containing mercury was also done. Psychiatry assessment stated that child is of sound mental health and of normal IQ. The skin rash and fever was transient and disappeared within a few days. Whole blood mercury levels reduced to normal and the child made an uneventful recovery and further follow up was arranged.

Although self-injection of elemental mercury is rare it is well documented [2], often as a part of suicide attempts or among drug users. Unusual instances are that of injection of mercury with the mistaken belief that it would strengthen sport performances [2]. Our patient was misguided by a movie and it was an experimental act. Deepthi Sukheeja et al. have reported similar case of a 15 year-old-male child who presented with multiple non healing ulcers of the left forearm due to self-injection of mercury after watching the same movie.

## Conclusion

Self-injection of elemental mercury can also occur in mentally sound people and rapid diagnosis and decontamination is required. This case also signifies the importance of imposing limitations for visual media which could misguide minors and lead those to imitate and cause serious self-harm. We suggest to include a warning saying “Injection or ingestion of mercury can cause serious bodily harm” at the start of this type of movies.

## Consent

Written informed consent was obtained from the parents for publication of this case report and any accompanying images. A copy of the written consent is available for review by the editor of this journal.
